# Long non-coding RNA MIAT promotes papillary thyroid cancer progression through upregulating LASP1

**DOI:** 10.1186/s12935-019-0913-z

**Published:** 2019-07-25

**Authors:** Wei Liu, Zhenglin Wang, Cong Wang, Zhilong Ai

**Affiliations:** Department of General Surgery, Zhongshan Hospital, Fudan University, 180 Fenglin Road, Shanghai, 200032 China

**Keywords:** MIAT, Papillary thyroid cancer, miR-324-3p, LASP1, Proliferation

## Abstract

**Background:**

Accumulating evidences indicate that long non-coding RNAs (lncRNAs) play an important role in initiation and development of thyroid cancer. However, the underlying molecular mechanism remains elusive.

**Methods:**

To explore potential oncogenic and tumor suppressive lncRNAs in papillary thyroid cancer (PTC), we performed RNA sequencing to discover differentially expression lncRNAs between PTC tissues and matched normal tissues. RT-qPCR was used to validate differentially expressed lncRNAs. Bioinformatic analysis was performed to predicted potential miRNA and gene which might be regulated by MIAT. Cell proliferation, invasion and cycle assay were conducted to study the function of MIAT and LASP1 in PTC.

**Results:**

Through analysis of RNA sequencing, we observed that lncRNA-MIAT was overexpressed in PTC tissues. The upregulation of MIAT was further confirmed in 40 pairs of PTC tissues and normal tissues we collected. In the function assays, results suggested that MIAT silencing led to inhibition of cell proliferation, invasion and disruption of cell cycle progression in PTC cells. Mechanistically, MIAT directly bound to miR-324-3p and upregulated LASP1 expression in PTC cells. In addition, expression of MIAT was positively correlated with LASP1 mRNA expression in samples collected from patients with PTC. More importantly, transfection of recombinant LASP1 attenuated MIAT silencing induced inhibition of cell proliferation, invasion and disruption of cell cycle progression in PTC cells.

**Conclusions:**

In conclusion, the findings suggest that lncRNA-MIAT may promote PTC proliferation and invasion through physically binding miR-324-3p and upregulation of LASP1.

**Electronic supplementary material:**

The online version of this article (10.1186/s12935-019-0913-z) contains supplementary material, which is available to authorized users.

## Background

Globally, thyroid cancer is the most commonly diagnosed endocrine malignancy, accounting for nearly 5% of new cancer cases [1]. Papillary thyroid cancer (PTC) is the most common type of thyroid cancer [2]. Patients with PTC have an excellent prognosis and more than half of the patients are curable [3]. However, there are still 30% of patients with aggressive PTC develop recurrence and distant metastasis, which would lead to patient death [4]. It is urgent to discover molecular mechanism of PTC to develop new effective therapeutic approaches so as to fulfil clinical needs.

Long non-coding RNAs (lncRNAs) are a class of transcripts with more than 200 nucleotides in length that are not translated into protein [5]. In recent years, studies reveal that a majority of lncRNAs are pivotal regulators of normal physiology processes [6, 7]. Dysregulation of lncRNAs are observed in many human diseases and contribute to disease progression [8–11]. Several lncRNAs were proved to play an important role during thyroid cancer carcinogenesis [12]. For example, lncRNA ANRIL inactivated TGF-β/Smad signaling pathway to reduce p15INK4B expression in thyroid cancer cells, led to enhanced cell invasion and metastasis ability [13]. LncRNA myocardial infarction associated transcript (MIAT) was firstly identified to be associated with a susceptibility to myocardial infarction [14]. Later, overexpression of MIAT was discovered in several types of cancers including neuroendocrine prostate cancer, breast cancer and chronic lymphocytic leukemias [15–17]. Mechanistically, previous studies showed that MIAT acted as a competing endogenous RNA (ceRNA) to physically bind miRNA and regulated gene expression [18]. However, it is still unknown whether and how MIAT contributes to PTC progression.

LIM and SH3 domain protein 1 (LASP1) is a member of LIM protein family which contains a LIM domain and two actin-binding domains [19]. In cells, LASP1 interacts with actin cytoskeleton and was reported to localized within sites of actin assembly [20]. In normal tissues, LASP1 is ubiquitously expressed and highly expressed in actin-rich tissues types [21]. In human tumor tissues, LASP1 is significantly overexpressed and plays a pivotal role for cancer aggressiveness [22]. The function assays suggested that LASP1 was involved in cell proliferation, migration and cycle regulation in cancer cells [23]. Most recently, a study showed that LASP1 was overexpressed in thyroid cancer and contributed to strong cell proliferation and migration ability of thyroid cancer cells through activation of PI3K/AKT pathway [24].

In this study, we discovered several differentially expressed lncRNAs between PTC tissues and normal tissues using RNA sequencing. We identified and confirmed MIAT as an upregulated lncRNA in PTC tissues compared with normal tissues. In the functional assays, our results showed that MIAT silencing decreased cell proliferation and invasion ability, disrupted cell cycle progression of PTC cells. Bioinformatic analysis and RT-qPCR defined a MIAT/miR-324-3p/LASP1 axis in PTC cells. The direct interaction among MIAT, miR-324-3p and LASP1 was verified with the dual luciferase reporter assay. Furthermore, rescue experiments suggested that MIAT regulated PTC cell proliferation, invasion and cycle rely on regulation of LASP1. These data demonstrated that MIAT might be a key lncRNA in promoting PTC progression.

## Materials and methods

### Collection of patient samples

A total of 40 pairs of PTC tissues and matched normal tissues were collected from patients who underwent surgical resection at Zhongshan Hospital, Fudan University during June 2015 to September 2017. No patients received chemotherapy or radiotherapy before surgery. The diagnosis of PTC was histopathological confirmed. All experiments were supervised by the Ethic Committee of Fudan University. Written consent was obtained from all participants before the study. The tissues were immediately frozen in liquid nitrogen then stored in − 80 °C for the following RNA extraction.

### RNA extraction, sequencing and expression quantification

RNA was extracted from 3 pairs of tissue samples using the Qiagen AllPrep DNA/RNA/Protein mini kit (Qiagen, Valencia, CA) according to the manufacturer’s protocol. After total RNA was extracted, rRNAs were removed to retain mRNAs and ncRNAs. The enriched mRNAs and ncRNAs were fragmented into short fragments by using fragmentation buffer and reverse transcribed into cDNA with random primers. Second-strand cDNA were synthesized by DNA polymerase I, RNase H, dNTP (dUTP instead of dTTP) and buffer. Next, the cDNA fragments were purified with QiaQuick PCR extraction kit (Qiagen), end repaired, poly(A) added, and ligated to Illumina sequencing adapters. Then UNG (Uracil-*N*-Glycosylase) was used to digest the second-strand cDNA. The digested products were size selected by agarose gel electrophoresis, PCR amplified, and sequenced using Illumina HiSeqTM 4000 by Gene Denovo Biotechnology Co. (Guangzhou, China). The reads of each sample were then mapped to reference genome by TopHat2 (version 2.1.1) and expression quantification was achieved using software RSEM [25]. We identified transcripts with a fold change ≥ 2 and a false discovery rate (FDR) < 0.05 in a comparison as significant DEGs.

### Cell culture

Papillary thyroid cancer cell lines HTH83 and BHT101 were bought from Cell Bank, Chinese Academy of Sciences (Shanghai, China). All cell lines were cultured in DMEM medium (Gibco, Rockville, MD) containing 10% FBS (Gibco) in a 37 °C incubator with 5% CO_2_.

### Bioinformatic analysis

The expression of lncRNAs in 9 pairs of papillary thyroid cancer tumor tissues and matched normal tissues from GSE3467 were downloaded from GEO database (https://www.ncbi.nlm.nih.gov/geo/). The expression of MIAT was analyzed using GEO2R online software. Expression data of MIAT and LASP1 in TCGA dataset (cell, 2014, containing expression data of 486 patients with PTC) were downloaded using cBioPortal [26]. The prediction of potential miRNA-mRNA interaction was carried out on miRDB V.5.0 (http://mirdb.org/). The expression of miR-324-3p and MIAT in 509 papillary thyroid cancer tissues from TCGA (The Cancer Genome Atlas) were analyzed on Starbase V.3.0 database (http://starbase.sysu.edu.cn/).

### RNA extraction and RT-qPCR

Total RNA was extracted from tissues and cells using TRIzol reagent (Invitrogen, Carlsbad, CA) following manufacturer’s protocol. The RNA was reverse transcribed into first stranded cDNA using ReverTra Ace-α qPCR RT Kit (Toyobo, Osaka, Japan). The qPCR was performed using SYBR Green Master Mix (Roche, Basel, Switzerland) on a CFX96 system (Bio-Rad, Hercules, CA). GAPDH and U6 were used as internal controls for mRNA and miRNA respectively. The relative expression of gene was calculated using 2^−ΔΔCT^ [27]. The primer sequences were listed in Table 1.

**Table 1:** Sequence of primers

Primer	Sequence
MIAT-F	5′-GCACCTTGAGTGAATGTCAAGGCAG-3′
MIAT-R	5′-TGGCAGCATCCAGCCGACACACAGG-3′
LASP1-F	5′-TGCGGCAAGATCGTGTATCC-3′
LASP1-R	5′-GCAGTAGGGCTTCTTCTCGTAG-3′
COLCA1-F	5′-CTTATGACAGGAAAGTGGAAG-3′
COLCA1-R	5′-TAGCATCAAGTTCCCATCCAC-3′
SHNG14-F	5′-TGCACAAAATAAGCCTGGCTGT-3′
SHNG14-R	5′-TCAATATTTAATACAGGCATGCA-3′
SHNG15-F	5′-TTCAGACAATGACTTCCTCCCTCCT-3′
SHNG15-R	5′-TAGCTCCTGGGGCACTCAGCTC-3′
RNU12-F	5′-TGCCTTAAACTTATGAGTAAGG-3′
RNU12-R	5′-GGGCCGGACTTATCTTTCTGAA-3′
LINC00667-F	5′-CTGAAATCACAGCAATGCCAGTTT-3′
LINC00667-R	5′-TATAGCTTTGATTTTCTTGCAGTGT-3′
GAPDH-F	5′-TTTGGTCGTATTGGGCGCCTGGTCA-3′
GAPDH-R	5′-TTGTGCTCTTGCTGGGGCTGGTGGT-3′
Stem-loop	5′-CTCAACTGGTGTCGTGGAGTCGGCAATTCAGTTGAGCCAGCA-3′
miR-324-3p-F	5′-GCCGAGCCCACTGCCCCAGG-3′
miR-324-3p-R	5′-CTCAACTGGTGTCGTGGA-3′
U6-F	5′-CTCGCTTCGGCAGCACA-3′
U6-R	5′-AACGCTTCACGAATTTGCGT-3′

### Protein extraction and western blotting

Protein lysates were prepared using RIPA lysis buffer (Beyotime, Shanghai, China). Antibodies against LASP1 (#8636, 1:1000) and GAPDH (#5174, 1:10,000) were purchased from Cell Signaling Technology (Lane Danvers, MA). Secondary antibodies for rabbit (#ab7090, 1:10,000) and mouse (#ab97040, 1:10,000) were obtained from Abcam (Cambridge, UK). 20 μg protein lysates were loaded into each lane on the 8% SDS-PAGE gel and then transferred into a PVDF membrane. The membrane was incubated with indicated primary antibody at 4 °C overnight. On the next day, the membrane was incubated with secondary antibody at room temperature for 1 h. The blot was developed with ECL Western Blotting Substrate (Pierce; Thermo Fisher Scientific, Waltham, MA).

### Silencing of MIAT

Control siRNA, MIAT siRNA1 and MIAT siRNA2 were synthesized and purchased from GenePharma (Shanghai, China). The sequences were: control siRNA:5′-UUCUCCGAACGUGUCACGUTT-3′; MIAT siRNA1:5′-GGUGUUAAGACUUGGUUUCTT-3′; MIAT siRNA2:5′-ACUUCUUCGUAUGUUCGGCTT-3′. For silencing of MIAT, MIAT siRNA1 or MIAT siRNA2 were mixed with Lipofectamine RNAiMax (Invitrogen) in 500 μL serum-free medium for 5 min, then added into culture medium. After 48 h, the cells were harvested and subjected to the following experiments.

### Overexpression and downregulation of miR-324-3p

MiR-NC mimic, miR-324-3p mimic, miR-NC inhibitor and miR-324-3p inhibitor were bought from GenePharma (Shanghai, China). MiRNA mimic or miRNA inhibitor was transfected into indicated cells using Lipofectamine RNAiMax (Invitrogen). After 48 h, the cells were harvested and subjected to the following experiments.

### Construction of plasmid and overexpression of LASP1

Full length of LASP1 open reading frame was amplified from HTH83 cDNA and ligated into pcDNA3. For overexpression of LASP1, pcDNA3-LASP1 plasmids were mixed with Lipofectamine 3000 (Invitrogen) in 500 μL serum-free DMEM medium for 15 min, then added into culture DMEM medium. After 48 h, the cells were harvested and subjected to the following experiments.

### Dual luciferase reporter assay

The 3′UTR of LASP1 was amplified from HTH83 cDNA and ligated into pGL3 plasmid. QuickChange Site-directed Mutagenesis Kit (Agilent Technologies, Santa Clara, CA) was used to introduce site mutations into LASP1 3′UTR-WT. For the dual luciferase reporter assay, cells were transfected with LASP1 3′UTR-WT or LASP1 3′UTR-Mut in combination with miR-NC mimic or miR-324-3p mimic using Lipofectamine 3000 (Invitrogen). After 48 h, the relative luciferase activity of each well was determined using Dual Luciferase Reporter System (Promega, Madison, WI).

### Cell invasion assay

Cell invasion assays were carried out using modified Boyden chambers consisting of Transwell (Corning Costar Corp., Cambridge, MA) membrane filter inserts in 24-well plates. The Transwell filters were 8 mm pore size polycarbonate membranes. The upper surfaces of the Transwell membranes were pre-coated with 1 mg/mL Matrigel (Becton–Dickinson Labware, Franklin Lakes, NJ) overnight at 4 °C then placed into 24-well plates. In each well, 500 μL culture medium was added. Cells (2 × 10^5^) in 100 μL of serum-free medium were added to each Transwell chamber and allowed to invade toward the underside of the membrane for 24 h. Cells in the upside of the chamber were removed, and the invaded cells were fixed and stained using 0.4% Crystal Violet Staining Solution (Solarbio, Beijing, China). The number of invaded cells per membrane was counted under a light microscope.

### Cell proliferation assay

The proliferation ability of cells was detected using Cell Counting Kit-8 (DoJinDo, Kumamoto, Japan) according to the manufacturer’s protocol. 1000 cells were seeded in each well in the 96-well plates. On the next day, cells were transfected with siRNA with or without plasmids. At the time point of 0, 24, 48, 72 h after treatment, 10 μL CCK-8 solution was added into each well and maintained for 1 h. The medium containing CCK-8 was then transferred into wells in a new 96-well plate and the absorbance at 450 nm was detected by a Microplate Reader (Bio-Rad) to reflect cell proliferation ability.

### Cell cycle assay

For cell cycle analysis, the cells were stained with Propidium Iodide (PI, Invitrogen). Briefly, after treatment, the cells were collected, washed with PBS and then fixed in 70% ethanol at 4 °C overnight. After that, PI was added into cell suspension and sustained for 30 min, after that, the cell distribution was analyzed on a flow cytometry (Becton–Dickinson Labware) with FlowJo software (Version 6.3.1, Tree Star Inc., Ashland, OH).

### Statistical analysis

All data analysis was carried out using Graphpad Prism 6.0 software. The values were presented as the mean ± SD. Differences between two groups were analyzed using Student’s t test. Differences from multiple groups were firstly analyzed with one-way ANOVA followed by Newman–Keuls analysis. A p value less than 0.05 was considered statistically significant.

## Results

### MIAT is highly expressed in papillary thyroid cancer (PTC) tissues and cells

**Fig. 1:**
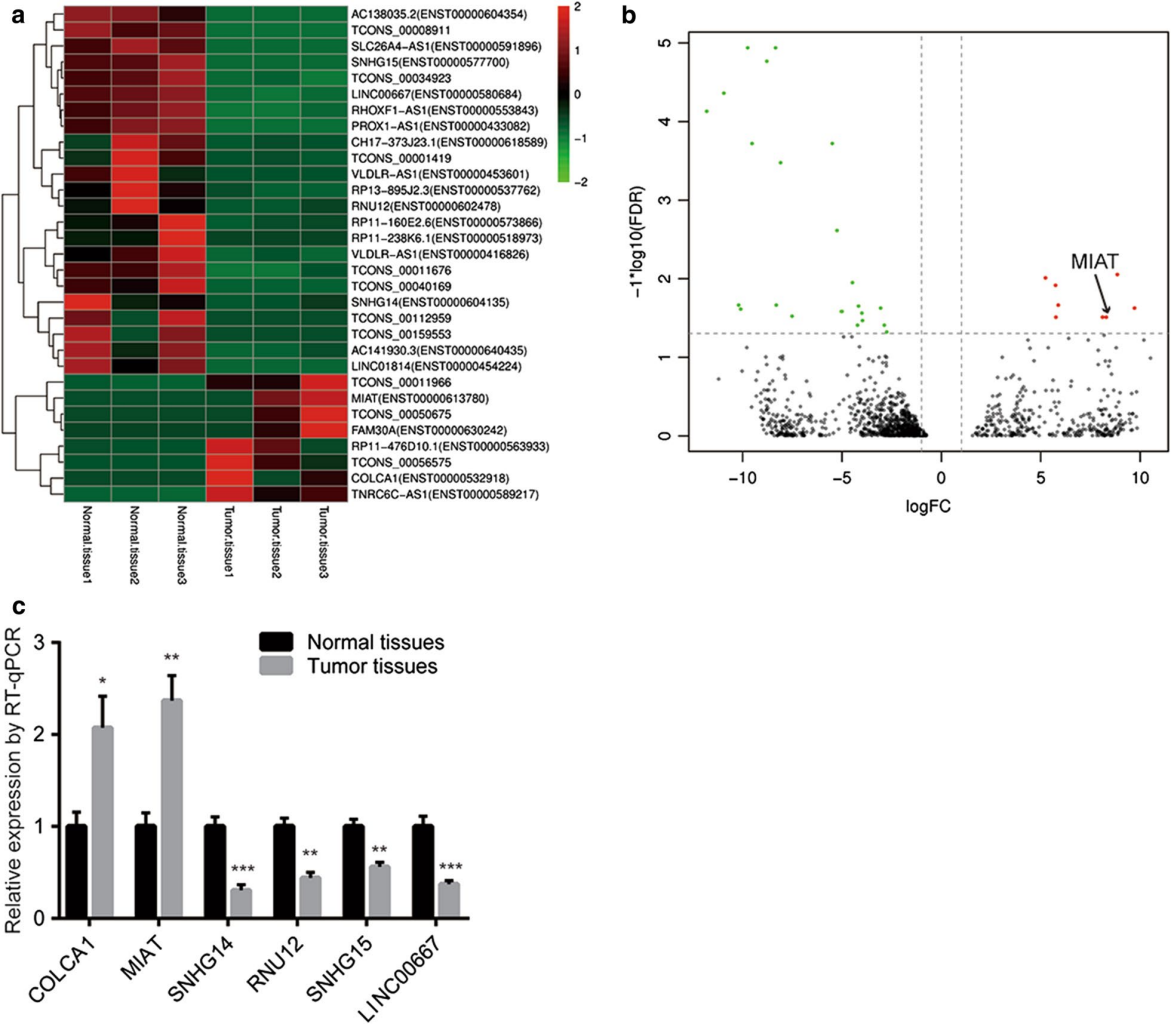
Screen of differentially expressed lncRNAs in papillary thyroid cancer. **a** Heatmap of differentially expressed lncRNAs (8 upregulated and 23 downregulated) in 3 papillary thyroid cancer tissues compared with matched normal tissues. **b** Volcano plots were constructed based on sequencing results of differentially expressed lncRNAs between papillary thyroid cancer tissues and normal tissues from 3 patients. The red points represent differentially upregulated genes, and green points represent downregulated genes. Arrow indicated MIAT in the plots. **c** RT-qPCR was performed to validate 6 differentially expressed lncRNAs (2 upregulated and 4 downregulated) obtained from sequencing of papillary thyroid cancer tissues and normal tissues of 3 patients. *p < 0.05; **p < 0.01; ***p < 0.001

**Fig. 2:**
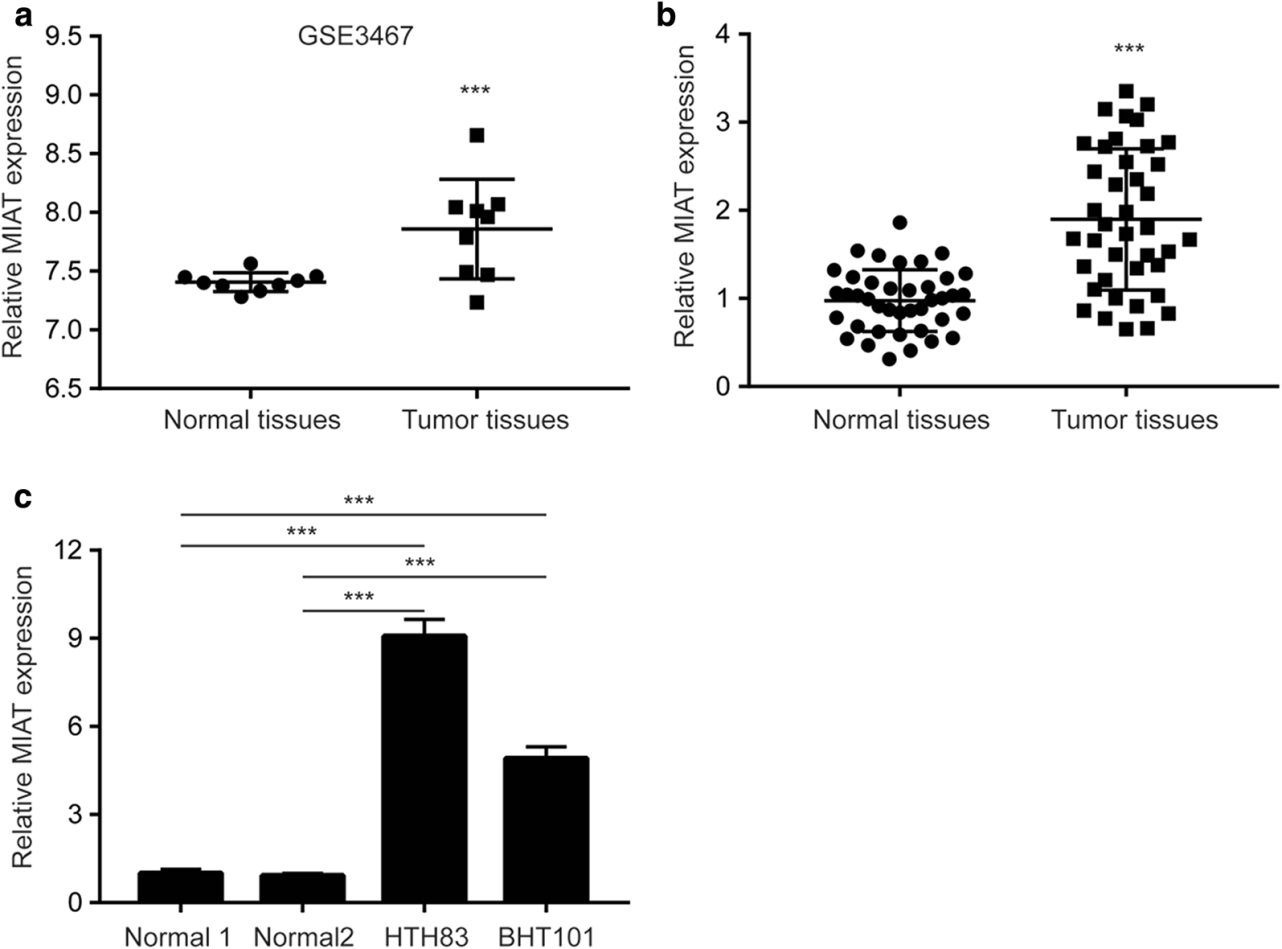
MIAT is overexpressed in papillary thyroid cancer and cells. **a** Bioinformatic analysis of GSE3467 dataset revealed that MIAT was overexpressed in papillary thyroid cancer compared with normal tissues from 9 patients. **b** RT-qPCR showed that MIAT was overexpressed in papillary thyroid cancer compared with normal tissues from 40 patients enrolled in this study. **c** RT-qPCR showed that MIAT was overexpressed in HTH83 and BHT101 cells compared with two normal thyroid tissues from patients with papillary thyroid cancer. ***p < 0.001

Using RNA sequencing method, we compared the expression of lncRNAs in three pairs of PTC tissues and normal tissues. The bioinformatic analysis revealed that several lncRNAs were differentially expressed (8 upregulated lncRNAs and 23 downregulated lncRNAs) between PTC tissues and normal tissues (Fig. 1a, b). Among them, most recent studies revealed that lncRNAs such as TNRC6C-AS1 were associated with progression of PTC [28, 29]. A total of 6 differentially expressed lncRNAs (COLCA1, MIAT, SNHG14, RNU12, SNHG15, LINC00667) were selected for validation in 10 pairs of PTC tissues and normal tissues using RT-qPCR. The data showed that these lncRNAs were differentially expressed between examined PTC tissues and normal tissues, and MIAT was significantly upregulated in PTC tissues (Fig. 1c). For further evaluation of our discovered differentially expressed lncRNAs in PTC, the array data in 9 pairs of PTC tissues and normal tissues were downloaded from GSE3467. The analysis indicated that TNRC6C-AS1 and MIAT expression were consistently increased in PTC tissues and MIAT was the most significantly upregulated lncRNA (Fig. 2a, Additional file 1: Figure S1). Moreover, in 40 pairs of PTC tissues and normal tissues we collected, overexpression of MIAT were also observed in PTC tissues (Fig. 2b). Additionally, it was observed that MIAT expression was significantly upregulated in HTH83 and BHT101, two PTC cell lines, compared with two normal thyroid tissues from patients with PTC (Fig. 2c). The data suggested that MIAT might promote PTC progression.

### MIAT promotes cell proliferation, cell cycle progression and invasion in PTC cells

**Fig. 3:**
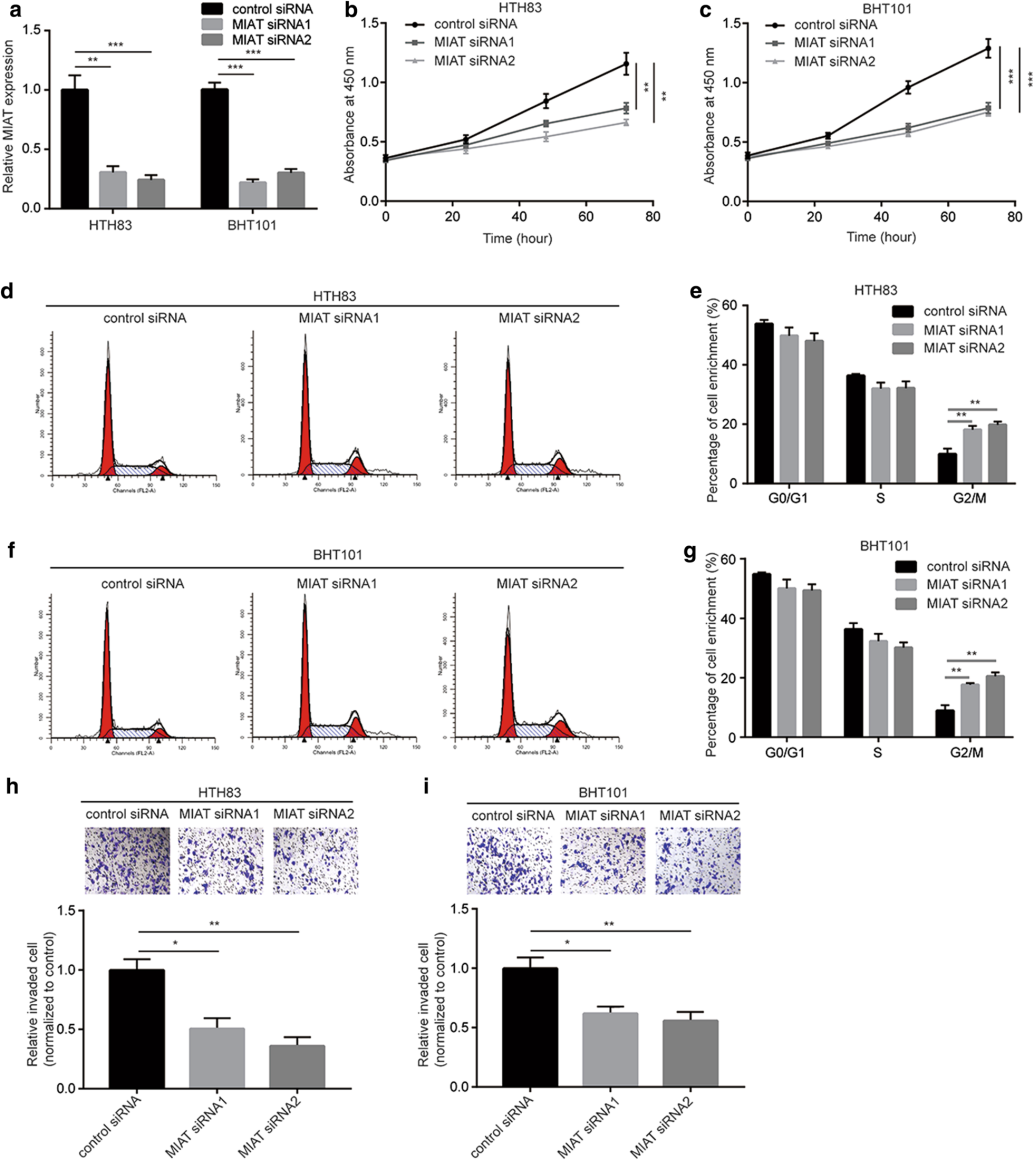
Silencing of MIAT inhibits cell proliferation, cycle and invasion ability of papillary thyroid cancer cells. **a** Transfection of MIAT siRNA1 or MIAT siRNA2 reduced MIAT expression in HTH83 and BHT101 cells. **b** Silencing of MIAT inhibited cell proliferation of HTH83 cells in a cell proliferation assay. **c** Silencing of MIAT inhibited cell proliferation of BHT101 cells in a cell proliferation assay. **d** The cell cycle analysis showed that MIAT silencing induced accumulation of HTH83 cells in G2/M phase. **e** Quantitative analysis of HTH83 cells enriched in G1, S and G2/M phase in **d**. **f** The cell cycle analysis showed that MIAT silencing induced accumulation of BHT101 cells in G2/M phase. **g** Quantitative analysis of BHT101 cells enriched in G1, S and G2/M phase in **f**. **h** Silencing of MIAT inhibited cell invasion of HTH83 cells in a cell invasion assay. **i** Silencing of MIAT inhibited cell invasion of BHT101 cells in a cell invasion assay. *p < 0.05; **p < 0.01; ***p < 0.001

To investigate the role of MIAT in PTC, MIAT was downregulated by transfection of two MIAT siRNAs in HTH83 and BHT101. Transfection of MIAT siRNA significantly downregulated MIAT expression in HTH83 and BHT101 cells (Fig. 3a). The cell proliferation assay data suggested that silencing of MIAT significantly inhibited the proliferation ability of both cell lines (Fig. 3b, c). Through the cell cycle analysis, we observed that MIAT downregulation increased cells accumulated in G2/M phase in HTH83 (Fig. 3d, e). Similar results were detected in BHT101 (Fig. 3f, g). In addition, it was found that the invasion capacity of HTH83 and BHT101 cells was greatly limited towards MIAT silencing (Fig. 3h, i). These results collectively manifested that MIAT was pivotal for proliferation and metastasis of PTC cells.

### MIAT acts as a competitive endogenous RNA (ceRNA) and directly binds to miR-324-3p in PTC cells

**Fig. 4:**
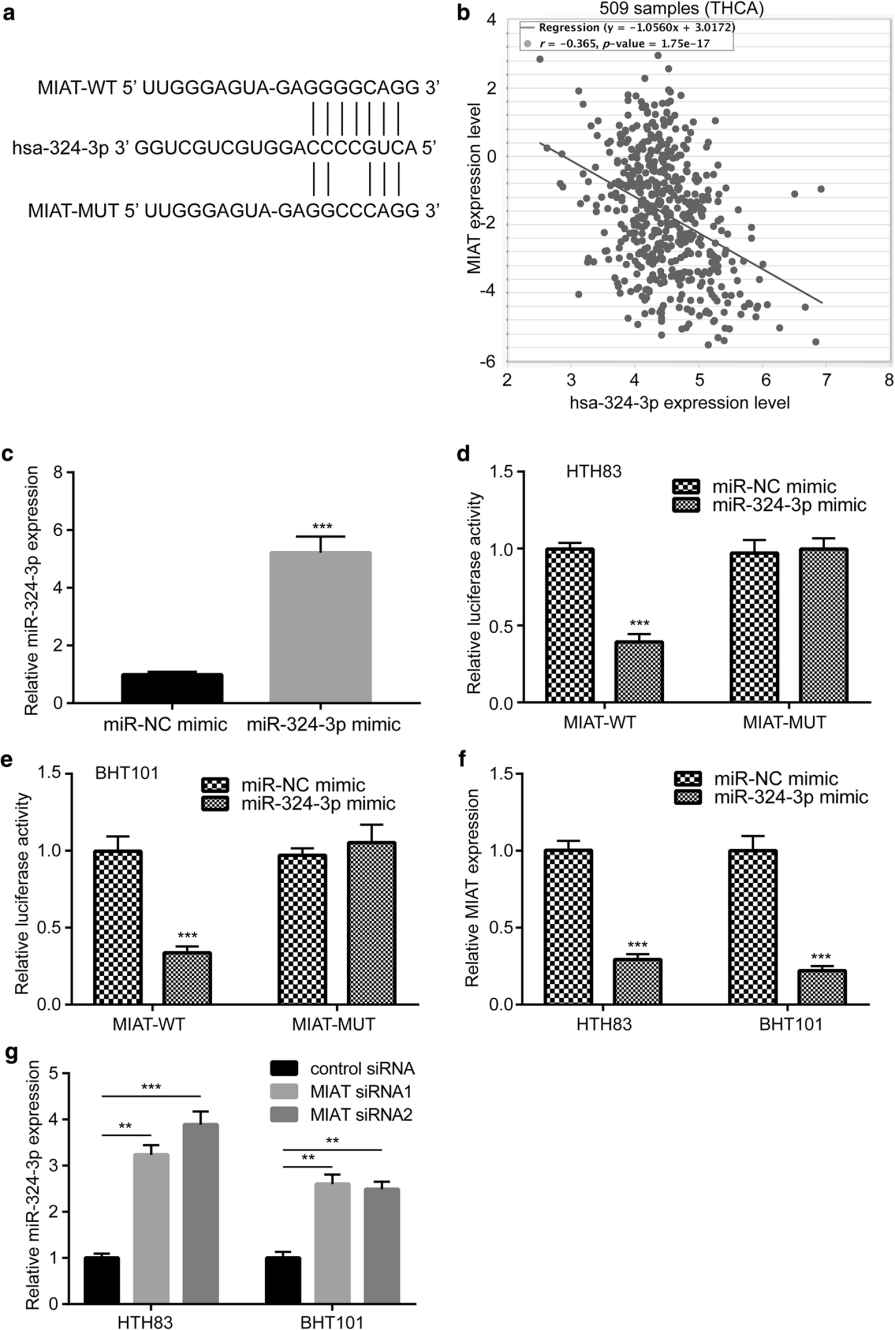
MIAT directly binds miR-324-3p in papillary thyroid cancer cells. **a** Sequencing alignment showed that miR-324-3p harbored putative binding site for MIAT. **b** Through analysis of gene expression data in 509 papillary thyroid cancer tissues from TCGA, a negative correlation was observed between miR-324-3p levels and MIAT levels. **c** Transfection of miR-324-3p mimic increased miR-324-3p expression in HTH83 cells. **d** Overexpression of miR-324-3p reduced relative luciferase activity of MIAT-WT not MIAT-MUT in HTH83 cells. **e** Overexpression of miR-324-3p reduced relative luciferase activity of MIAT-WT not MIAT-MUT in BHT101 cells. **f** Overexpression of miR-324-3p reduced miR-324-3p levels in HTH83 and BHT101 cells. **g** Silencing of MIAT increased miR-324-3p levels in HTH83 and BHT101 cells. **p < 0.01; ***p < 0.001

Using Starbase V.3.0 database, we predicted several potential miRNAs which might bind to MIAT. Among them, miR-324-3p harbors a potential binding site for MIAT (Fig. 4a) and its expression is negatively correlated (r = − 0.365, p < 0.000) with MIAT levels in expression data of 509 thyroid carcinoma tissues (THCA) from TCGA (Fig. 4b). In the dual luciferase assay, overexpression of miR-324-3p significantly reduced relative luciferase activity of MIAT-WT in both HTH83 and BHT101 cells while the relative luciferase activity of MIAT-MUT (with two site mutations in potential binding sequence) was not altered in both cell lines (Fig. 4c–e). Overexpression of miR-324-3p downregulated MIAT expression in HTH83 and BHT101 cells (Fig. 4f). Conversely, MIAT silencing increased miR-324-3p levels in these two cell lines (Fig. 4g).

### MIAT regulates LASP1 expression via sponging miR-324-3p in PTC cells

**Fig. 5:**
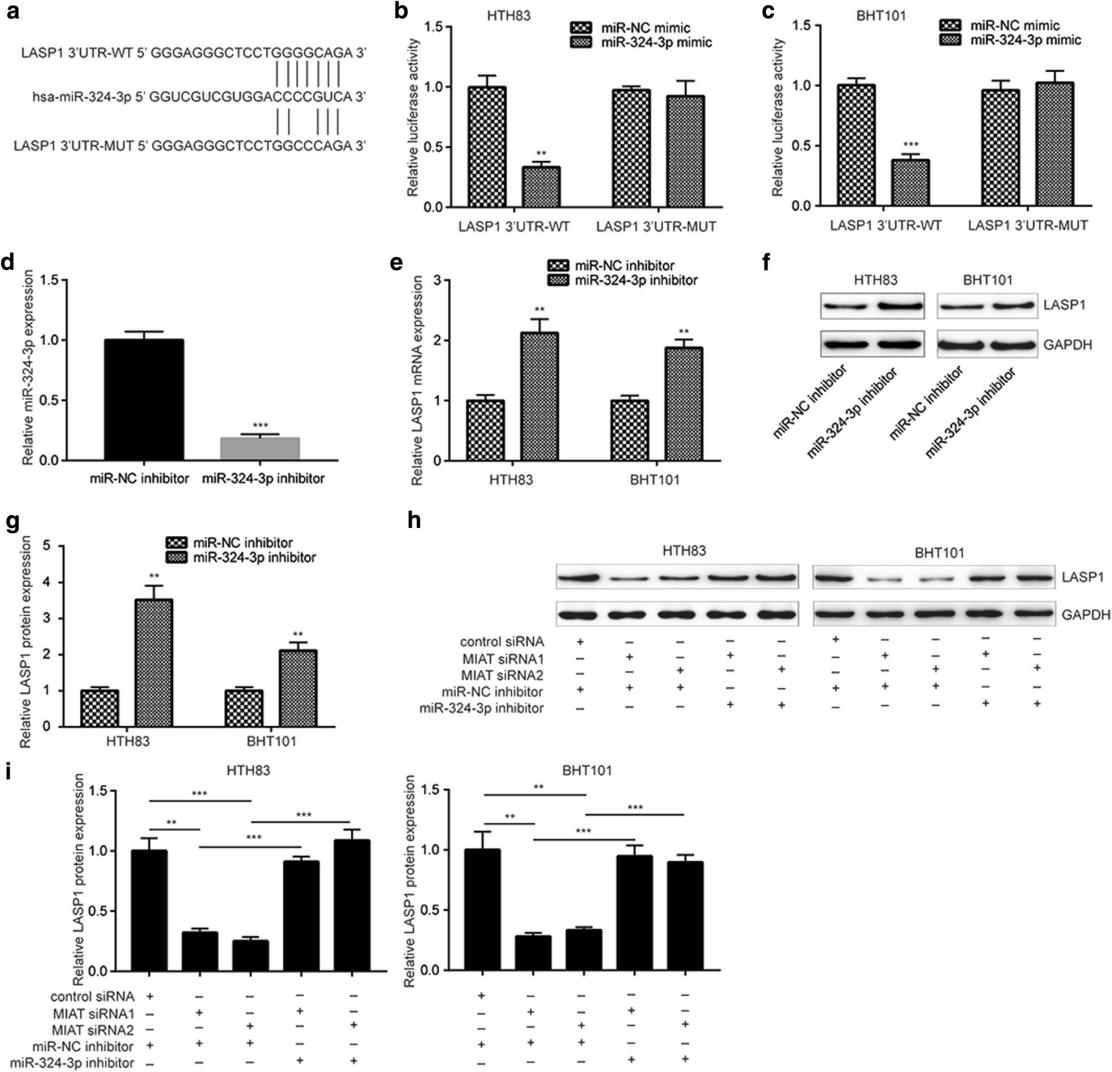
MIAT regulates LASP1 expression via miR-324-3p in papillary thyroid cancer cells. **a** There was a putative binding site for miR-324-3p on the 3′UTR of LASP1 mRNA. **b** Overexpression of miR-324-3p reduced relative luciferase activity of LASP1 3′UTR-WT not LASP1 3′UTR-MUT in HTH83 cells. **c** Overexpression of miR-324-3p reduced relative luciferase activity of LASP1 3′UTR-WT not LASP1 3′UTR-MUT in BHT101 cells. **d** Transfection of miR-324-3p inhibitor decreased miR-324-3p levels in HTH83 cells. **e** Downregulation of miR-324-3p increased mRNA levels of LASP1 in both HTH83 and BHT101 cells. **f** Western blotting showed that downregulation of miR-324-3p increased protein levels of LASP1 in both HTH83 and BHT101 cells. **g** Quantitative analysis of protein expression in **f**. **h** Western blotting showed that silencing of MIAT decreased LASP1 expression which was reversed towards downregulation of miR-324-3p in both HTH83 and BHT101 cells. **i** Quantitative analysis of protein expression in **h**. **p < 0.01; ***p < 0.001

Using miRDB database, LASP1 was predicted to be a potential target gene of miR-324-3p (Fig. 5a). Overexpression of miR-324-3p reduced relative luciferase activity of LASP1 3′UTR-WT not LASP1 3′UTR-MUT (with two site mutations in potential binding sequence) in HTH83 and BHT101 cells (Fig. 5b, c). In both HTH83 and BHT101, transfection of miR-324-3p inhibitor increased LASP1 mRNA levels (Fig. 5d, e). Western blotting further showed that the protein levels of LASP1 was also increased towards miR-324-3p downregulation (Fig. 5f, g). More importantly, we found that silencing of MIAT decreased LASP1 protein levels which was reversed after co-transfection of miR-324-3p inhibitor in the two cell lines (Fig. 5h, i). The data suggested that MIAT, miR-324-3p and LASP1 acted together in a ceRNA mechanism.

### The MIAT/miR-324-3p/LASP1 axis determines the cell proliferation, cycle and invasion of PTC cells

**Fig. 6:**
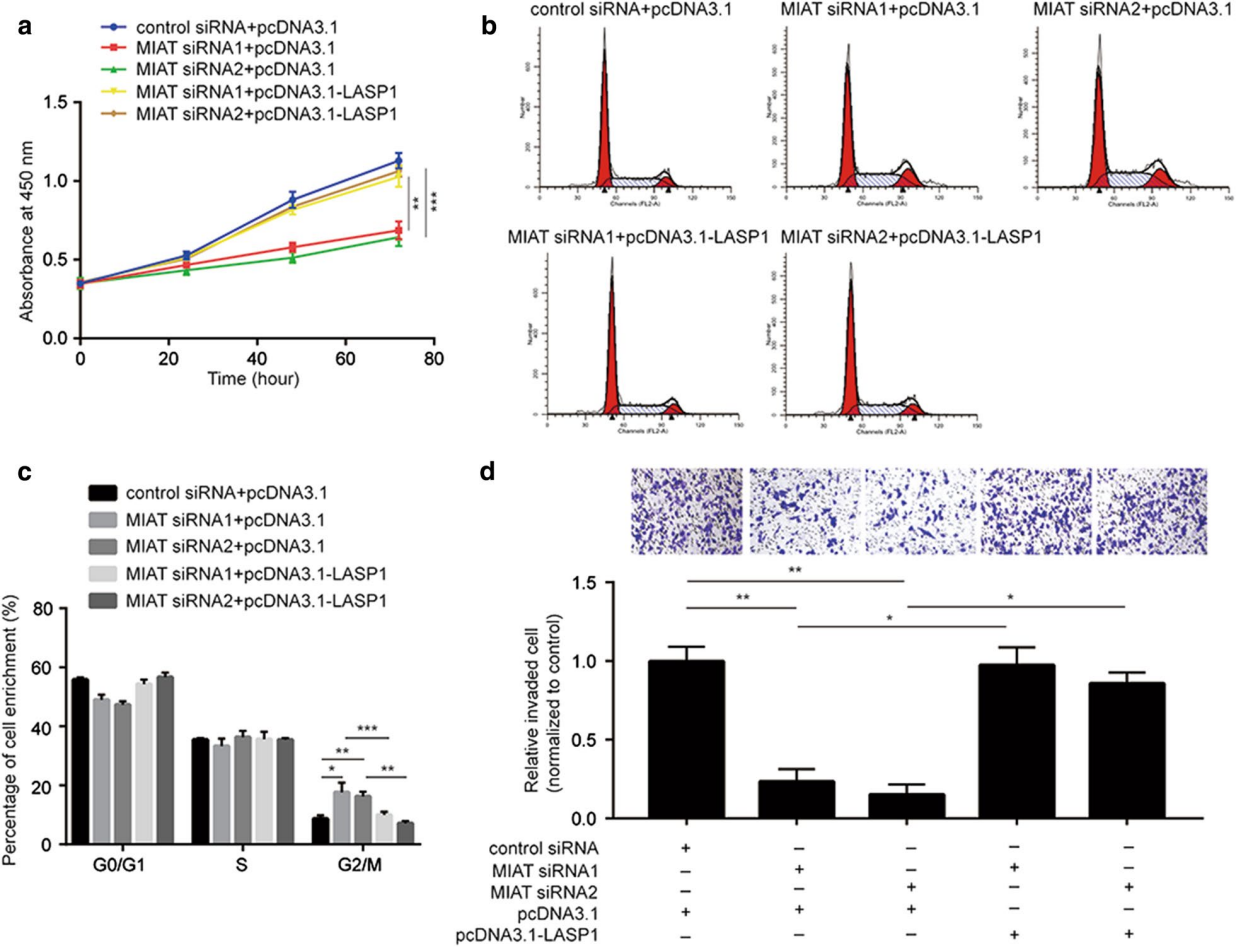
Overexpression of LASP1 reverses MIAT silencing induced inhibition of cell proliferation, cycle and invasion in papillary thyroid cancer cells. **a** MIAT silencing inhibited cell proliferation which was reversed after transfection of recombinant LASP1 in HTH83. **b** MIAT silencing induced accumulation of cells in G2/M phase which was reversed after transfection of recombinant LASP1 in HTH83. **c** Quantitative analysis of cell cycle distribution in **b**. **d** MIAT silencing inhibited cell invasion ability which was reversed after transfection of recombinant LASP1 in HTH83. *p < 0.05; **p < 0.01; ***p < 0.001

To investigate whether the new identified MIAT/miR-324-3p/LASP1 axis was involved in development of PTC, we performed functional assays in cells transfected with MIAT siRNA in combination with recombinant LASP1. As showed in Fig. 6a, overexpression of LASP1 reversed MIAT downregulation induced cell growth arrest. In addition, LASP1 overexpression attenuated MIAT silencing induced cell accumulation at G2/M phase (Fig. 6b, c). Moreover, LASP1 overexpression also reversed reduced cell invasion ability led by MIAT silencing (Fig. 6d). The data collectively manifested that MIAT regulated PTC development via regulation of LASP1.

### LASP1 expression is positively correlated with MIAT in PTC tissues

**Fig. 7:**
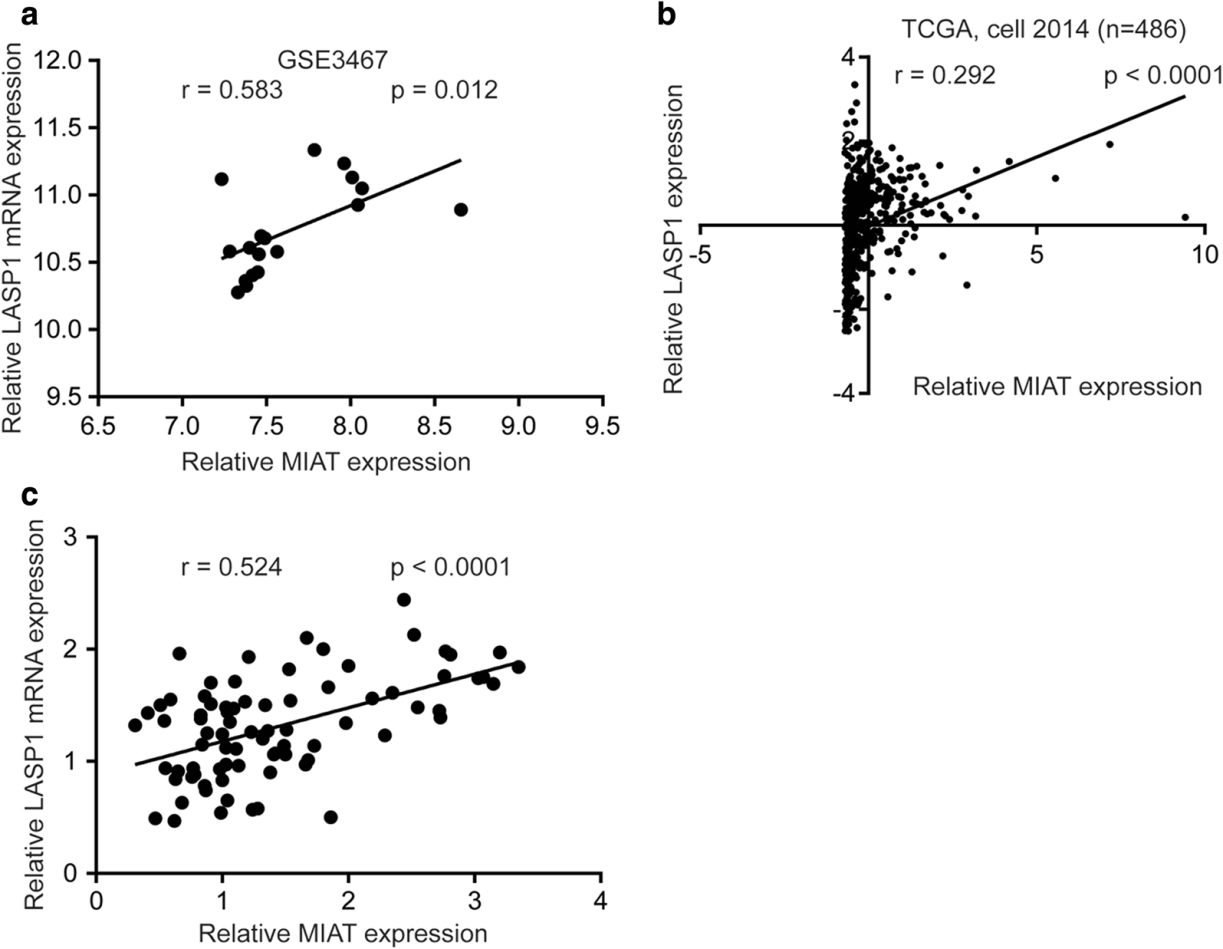
MIAT expression is correlated with LASP1 mRNA levels in papillary thyroid cancer tissues. **a** Through analysis of expression data from 9 PTC tissues in GSE3467, a significant positive correlation between LASP1 mRNA levels and MIAT levels was observed in tumors and normal tissues from 9 patients with PTC. **b** Via analysis of TCGA dataset (cell, 2014), a significant positive correlation between LASP1 mRNA levels and MIAT levels was observed in tumors from 486 patients with PTC. **c** A significant positive correlation between LASP1 mRNA levels and MIAT levels was observed from 40 PTC tissues we collected

For further examine the clinical association between LASP1 and MIAT expression, expression data of 9 pairs of PTC tissues and normal tissues from GSE3467 was analyzed. As we expected, a positive correlation (r = 0.583, p < 0.05) between their expression was observed (Fig. 7a). Furthermore, in a large cohort (TCGA, cell 2014), expression of MIAT was positively correlated with LASP1 levels in tumor from 486 patients with PTC (r = 0.292, p < 0.0001) (Fig. 7b). In the validation group (40 pairs of PTC tissues and normal tissues we collected), we also observed a positive correlation (r = 0.524, p < 0.001) between LASP1 mRNA levels and MIAT levels (Fig. 7c).

## Discussion

In PTC, several lncRNAs have already been identified as oncogenes or tumor suppressors through regulating key genes involved in cell proliferation and migration. In addition, some lncRNAs are proved to be excellent prognostic predictors for patients with PTC. In the present study, we firstly identified several differentially expressed lncRNAs between PTC tissues and normal tissue via sequencing. Among them, aberrant expression of SNHG14 and SNHG15 have been discovered in cancers [30, 31], expression of LINC00667 was proved as a biomarker to predict relapse free survival in patients with small hepatocellular carcinoma [32], most recently, the roles of VLDLR-AS1 and TNRC6C-AS1 in PTC have been studied [33, 34]. In the current study, we revealed a pivotal role of MIAT in regulation of PTC progression.

The overexpression of MIAT in tumor tissues has been reported in several cancer types [18, 35]. Using RNA-seq method, we discovered that MIAT was one of the most significantly elevated lncRNAs in PTC tumor tissues compared with matched normal tissues. Functional assays suggested that MIAT played a key role in regulation of cell proliferation, invasion and cycle of PTC cells. Known as miRNA “sponge”, competing endogenous RNAs (ceRNAs) are RNA transcripts that compete for binding to miRNA through the base-pairing method, which leads to reduction of miRNAs available to target mRNA [36]. Recent studies found that MIAT was involved in cancer progression via sponging miRNAs [37]. For example, in colorectal cancer cells, MIAT bound to miR-132, a tumor suppressor in colorectal cancer, led to downregulation of miR-132 and facilitate proliferation and metastasis of cancer cells [38]. Through bioinformatic analysis, we observed that MIAT might bind to miR-324-3p. MiR-324-3p was reported to be a tumor suppressor in nasopharyngeal carcinoma [39]. Moreover, the downregulation of miR-324-3p was discovered in many cancer types [40]. Our data showed that miR-324-3p was directly targeted by MIAT, suggested that MIAT might promote cancer progression via miR-324-3p.

LASP1 was a recently discovered oncogene in PTC [24]. Silencing of LASP1 inactivated PI3 K/AKT signaling and induced cell proliferation and migration inhibition in PTC cells [24]. Previous studies manifested that dysregulation of microRNAs led to LASP1 overexpression in cancer cells [41, 42]. In gastric cancer, hypermethylation of DNA led to downregulation of miR-29b, which contributed to overexpression of LASP1 [43]. We found that there was a putative binding site on LASP1 3′UTR for miR-324-3p. Further experiments validated that LASP1 was a direct target gene of miR-324-3p. This suggested a regulatory association among MIAT, miR-324-3p and LASP1. Indeed, silencing of MIAT decreased LASP1 expression in PTC cells. More importantly, analysis of PTC expression dataset on public database and PTC tissues we collected indicated that there was a positive correlation between MIAT and LASP1 expression. LASP1 was proved to be regulated by several lncRNAs (PVT1, AFAP1-AS1) [44, 45], our study further identified MIAT as a new regulator of LASP1, at least in PTC cells. In the functional assays, overexpression of LASP1 recovered MIAT silencing induced cell proliferation, invasion and cycle inhibition, suggested that MIAT relied on regulation of LASP1 to regulate PTV cancer progression. Thus, a MIAT/miR-324-3p/LASP1 axis was discovered as a driver of PTC progression.

## Conclusion

In conclusion, our data identified lncRNA MIAT as a key lncRNA in promoting the pathogenesis of PTC. MIAT might be used as potential biomarker or therapeutic target in the future, and should be the focus of future studies into the molecular mechanism of PTC.

## Additional file

**Additional file 1: Figure S1.** Analysis of COLCA1, TNRC6C-AS1 and FAM30A expression in normal and tumor tissues from patients with PTC. A. Bioinformatic analysis of GSE3467 dataset revealed that there was no significant difference between COLCA1 expression in papillary thyroid cancer compared with normal tissues from 9 patients. B. Bioinformatic analysis of GSE3467 dataset revealed that TNRC6C-AS1 was overexpressed in papillary thyroid cancer compared with normal tissues from 9 patients. C. Bioinformatic analysis of GSE3467 dataset revealed that there was no significant difference between FAM30A expression in papillary thyroid cancer compared with normal tissues from 9 patients.

## Data Availability

They are available under special request.
