# Supplementary figures and images for: Long non-coding RNA MIAT promotes papillary thyroid cancer progression through upregulating LASP1
Source: Cancer Cell Int. 2019 Jul 25;19:194. doi: 10.1186/s12935-019-0913-z (PMC6659215; doi:10.1186/s12935-019-0913-z)

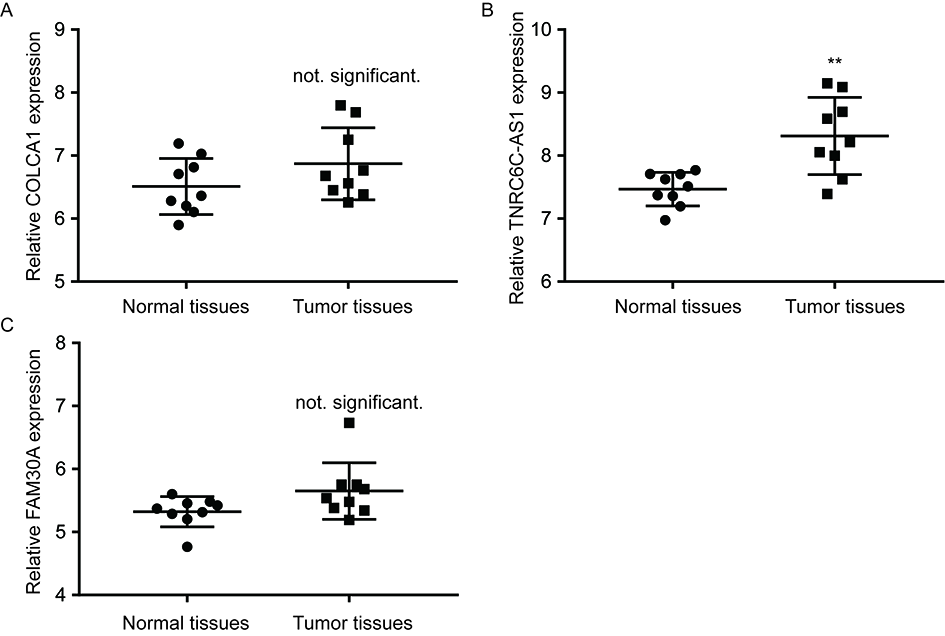

Supplement: Supplementary file 1 — Additional file 1: Figure S1. Analysis of COLCA1, TNRC6C-AS1 and FAM30A expression in normal and tumor tissues from patients with PTC. A. Bioinformatic analysis of GSE3467 dataset revealed that there was no significant difference between COLCA1 expression in papillary thyroid cancer compared with normal tissues from 9 patients. B. Bioinformatic analysis of GSE3467 dataset revealed that TNRC6C-AS1 was overexpressed in papillary thyroid cancer compared with normal tissues from 9 patients. C. Bioinformatic analysis of GSE3467 dataset revealed that there was no significant difference between FAM30A expression in papillary thyroid cancer compared with normal tissues from 9 patients. [file 12935_2019_913_MOESM1_ESM.tif]
